# Prostaglandin E_2_ and the Suppression of Phagocyte Innate Immune Responses in Different Organs

**DOI:** 10.1155/2012/327568

**Published:** 2012-09-13

**Authors:** Alexandra Medeiros, Camila Peres-Buzalaf, Felipe Fortino Verdan, C. Henrique Serezani

**Affiliations:** ^1^Department of Biological Sciences, School of Pharmaceutical Sciences, São Paulo State University (UNESP), Araraquara, 14801-902 São Paulo, SP, Brazil; ^2^Department of Biological Sciences, Bauru School of Dentistry, University of São Paulo (USP), Av Octávio Pinheiro Brisolla 9-75, Bauru, 17012-901 São Paulo, SP, Brazil; ^3^Department of Microbiology and Immunology, Indiana University School of Medicine, 950 West Walnut Street, Indianapolis, IN 46202, USA

## Abstract

The local and systemic production of prostaglandin E_2_ (PGE_2_) and its actions in phagocytes lead to immunosuppressive conditions. PGE_2_ is produced at high levels during inflammation, and its suppressive effects are caused by the ligation of the E prostanoid receptors EP_2_ and EP_4_, which results in the production of cyclic AMP. However, PGE_2_ also exhibits immunostimulatory properties due to binding to EP_3_, which results in decreased cAMP levels. The various guanine nucleotide-binding proteins (G proteins) that are coupled to the different EP receptors account for the pleiotropic roles of PGE_2_ in different disease states. Here, we discuss the production of PGE_2_ and the actions of this prostanoid in phagocytes from different tissues, the relative contribution of PGE_2_ to the modulation of innate immune responses, and the novel therapeutic opportunities that can be used to control inflammatory responses.

## 1. General Considerations

**Figure 1:**
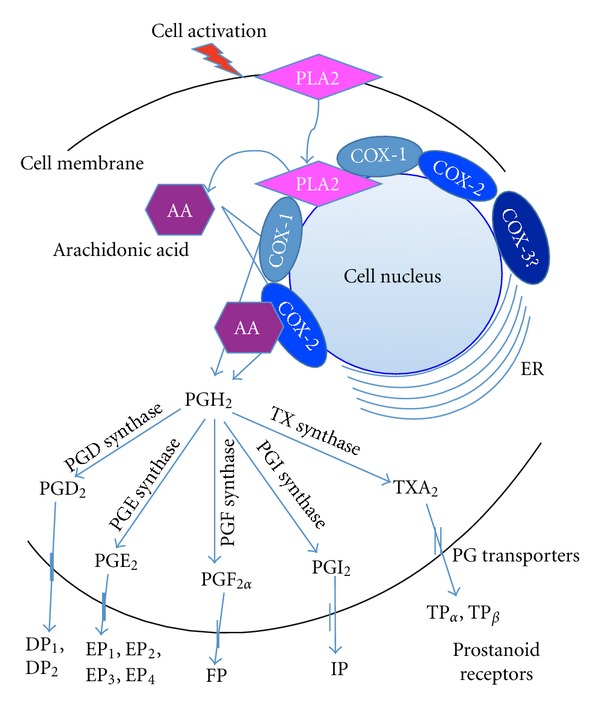
Prostanoid biosynthesis and receptors. Upon cell stimulation, PLA2 is activated, and (AA) is released from the cellular membranes. AA is then metabolized by COX-1 or COX-2 in different cellular compartments and further metabolized by different synthases, which leads to the generation of different prostanoids. Once the product is formed, different prostanoids are transported outside the cells to bind to their respective receptors. (PG prostaglandin; Tx thromboxane; PGJ_2_ 15-deoxy-Δ^12,14^-prostaglandin J_2_; Cox-1/2 cyclooxygenase-1/2; PGDS, PGES, PGFS, and PGIS prostaglandin D_2_/E_2_/F_2_/I_2_-synthase; PGIS prostacyclin synthase; TxAS thromboxane A_2_ synthase; PGER prostaglandin E2 9-reductase).

Prostaglandins (PGs) are lipid mediators derived from arachidonic acid (AA) metabolism via the activation of the cyclooxygenase (COX) pathway, that regulates inflammation, immune response, hematopoiesis, tissue injury and repair, and bone resorption. PGs are found in most tissues and organs, and the variety of effects that they can elicit reflects the presence of specific PG receptors in many cell types. Upon cell activation by microbial products, cytokines, and opsonins, cytosolic phospholipase A_2_ (PLA_2_) is activated and recruited to hydrolase plasma cell phospholipids. Once it is released from the membrane, AA is rapidly converted into PGs by cells expressing prostaglandin H synthase (COX). At least two COX isoforms exist, the constitutive (COX-1) and inducible (COX-2) isoforms. COX-1 is expressed in many cell types distributed throughout the body, whereas COX-2 expression is highly restricted under basal conditions and upregulated during inflammation in different cell types [1] (see Figure 1). COX proteins are the major targets of nonsteroidal anti-inflammatory drugs (NSAIDs).

COX-2 is transcriptionally regulated by mediators that act through phosphatidylinositol 3-kinase (PI3K), extracellular signal-regulated kinase1/2 (ERK1/2), and p38, and the activation of COX-2 culminates in the activation of the transcription factors, nuclear factor kappa B (NF*κ*B), activator protein (AP-1) and the cAMP response element-binding (CREB) [2, 3]. Therefore, COX-2 activity is induced by a variety of proinflammatory cytokines and growth factors and by one of its products, PGE_2_. Conversely, COX-2 expression is inhibited by glucocorticoids and interleukin (IL)-4. Both COX-1 and COX-2 are present in the active state in the endoplasmic reticulum and the nuclear envelope. These enzymes convert AA to the unstable endoperoxide PGH_2_, which is converted by specific synthases to the five following biologically active prostanoids: PGD_2_, PGE_2_, PGF_2*α*_, PGI_2_ (prostacyclin), and thromboxane A2 (TXA_2_). There are several PGE synthases, and one of these synthases (mPGES-1) is a highly inducible microsomal enzyme that acts downstream of COX to catalyze the conversion of PGH_2_ to PGE_2_ [4–6] (Figure 1).

**Figure 2:**
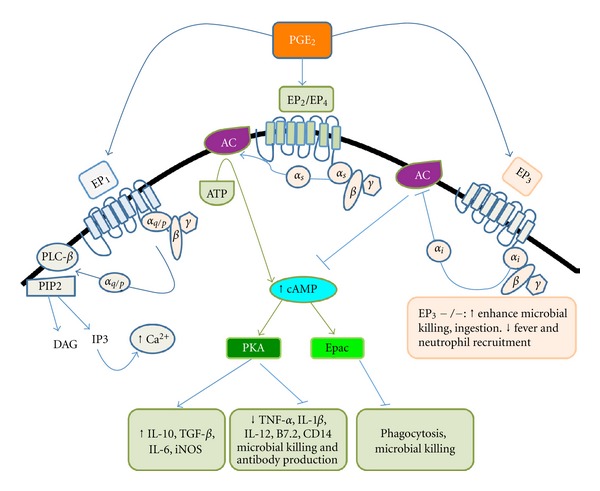
PGE_2_ receptors and their actions in macrophages. PGE_2_ produced during inflammatory conditions binds to EP_2_, EP_4_, EP_3_, or EP_1_. EP_2_ and EP_4_ are coupled to G*α*_*s*_, and the binding of PGE_2_ to these G protein-coupled receptors (GPCRs) induces a conformational change that results in the liberation of the G*α*_*s*_ subunit from the G*βγ* subunit complex. The binding of the G*α* subunit to adenylyl cyclase (AC) either stimulates (G*α*_s_) or inhibits (G*α*_i_, via EP_3_ signaling) the enzyme's generation of cAMP. The production of cAMP is also regulated by microbial pathogens. Downstream cAMP signaling is mediated by its interactions with effector molecules, such as protein kinase A (PKA), or exchange proteins that are directly activated by cAMP (Epac), which have been shown to modulate phagocyte functions. Depicted here is a pattern for alveolar macrophages in which specific antimicrobial functions are differentially regulated by specific cAMP effectors.

PGE_2_ is a potent mediator of inflammation that induces both pro- and anti-inflammatory effects and signals via four different E prostanoid (EP) receptors, EP_1_-EP_4_. The EP receptors are member of a family of G protein-coupled receptors (GPCRs). EP_1_ signals through G*α*_q_, which leads to increased levels of Ca^2+^. EP_2_ and EP_4_ signal through G*α*_s_, which leads to increased cAMP levels. EP_3_ primarily signals through G*α*_i_, which leads to decreased cAMP levels [7] (Figure 2).

The distribution and relative expression of these four receptor subtypes provide an elegant system that can account for the ability of PGE_2_ to evoke pleiotropic and sometimes opposing bioactions that are tissue- and cell-type specific.

Although PGE_2_ is commonly considered to be a potent proinflammatory mediator [8], its role as a mediator of anti-inflammatory responses is now being studied [9, 10]. The anti-inflammatory response opposes the host inflammatory response, which potentially limits collateral damage to neighboring cells and tissues and aids in the resolution of inflammation after the pathogens are contained [11]. This dual effect depends on the cell type, the tissue compartment, the state of cellular activation, and the particular expression of the signaling-EP receptors. The existence of four subtypes of receptors that signal differently and can be expressed in different combinations in a single cell explains the multiplicity of biological responses that are elicited by PGE_2_ and how these responses may differ among cells and tissues. This paper reviews the recent knowledge regarding PGE_2_ synthesis and its modulatory effect on innate immune responses in different tissues.

## 2. Lung

The synthesis of PGE_2_ occurs in several different cellular types within the airways, such as epithelial cells, fibroblasts, vascular endothelial cells, and leukocytes [12]. The leukocytes that can synthesize PGE_2_ include the alveolar macrophages (AMs), neutrophils, follicular dendritic cells, and T cells. The relative capacity of these cells to produce PGE_2_ is shown in Table 1. The AMs represent a major source of PGE_2_ during microbial infection [13], whereas alveolar epithelial cells and pulmonary fibroblasts also represent an important source of PGE_2_ in the lungs [14]. High levels of PGE_2_ are produced in AMs following the lipopolysaccharide (LPS)-and granulocyte/macrophage colony-stimulating factor (GM-CSF)-dependent expression of the inducible form of COX-2 [15]. Several mediators and signal transduction pathways are involved in the modulation of the synthesis and release of PGE_2_ by these cells. The inhibition of endogenous rat AM-producing transforming growth factor (TGF)-*β* enhances PGE_2_ synthesis, while the expression of LPS-induced COX-2 and PGE_2_, which are released by human AMs, is upregulated following the inhibition of PI3K activity [3]. AMs also produce increased PGE_2_ after bone marrow transplantation [16]. Although neutrophils are considered to be the main producers of leukotriene B_4_ (LTB_4_) (5-lipoxygenase-derived lipid mediator), few studies have attempted to evaluate the ability of lung neutrophils to produce prostanoids. In fact, the majority of studies is focused on the peritoneal and peripheral blood-derived neutrophils [17]. One of these studies demonstrated that lung PMNs (but not AMs) from mice that received bone marrow transplants synthesized pronounced levels of PGE_2_ when compared with cells from control mice [16]. In general, the *in vitro *synthesis of the cytokine-induced PGE_2_ by neutrophils involves the activation and novel synthesis of COX [18]. In addition, while PGE_2_ synthesis is well documented in human monocyte-derived immature dendritic cells (DCs) [19], no studies to date have demonstrated the particular capacity of lung DCs to produce this mediator.

PGE_2_ produced in the lungs elicits a wide variety of effects [1]. The effects vary from the induction of tissue repair and pulmonary vascular remodeling [20] to the regulation of immune inflammatory responses [21].

AMs are the primary lung cells that are involved in the protection of the alveolar-blood interface and serve as the front line of cellular defense against respiratory pathogens [22] in both murine and human cells. AMs express all four types of EP receptors [23] and contribute greatly to the amount of PGE_2_ produced in infected lungs [13] (Table 1). Monick and collaborators have demonstrated that LPS induces COX-2 expression and PGE_2_ release in human AMs [3, 24].

The immunomodulatory effects of PGE_2_ are largely caused by its ability to increase intracellular cAMP through the stimulatory G*α*_s_-coupled EP receptors EP_2_ and EP_4_ [25]. Increases in intracellular cAMP levels are transduced into cellular responses mediated by its effectors, cAMP-dependent protein kinase A (PKA), and the exchange protein directly activated by cAMP-1 (Epac-1) [26]. In phagocytes, the effects of PGE_2_ are usually anti-inflammatory since PGE_2_ has been demonstrated to inhibit the production of proinflammatory molecules and increase the secretion of anti-inflammatory cytokines, such as IL-10 [27]. In human AMs, PGE_2_ potently inhibited LPS-induced tumor necrosis factor (TNF)-*α* through the activation of the EP_2_ and EP_4_ receptors [28]. The downmodulation of LPS-induced TNF-*α* by PGE_2_ in rat AMs is dependent on cAMP signaling-dependent PKA activation since the selective PKA activating cAMP analog 6-Bnz-cAMP, but not the Epac-1 activating analog 8-pCPT-2-O-Me-cAMP, inhibits its production [29]. EP_2_ signaling is also involved in the enhancement of LPS-induced nitric oxide (NO) by the activation of PKA rather than Epac-1 [30]. Exogenous PGE_2_ can potentiate the synthesis of LPS-mediated IL-6 and IL-10 in rat AMs via AKAP10-(A-kinase anchoring protein-10-) mediated PKA signaling, while the suppression of TNF-*α* occurs via AKAP-8-anchored PKA-RII (PKA regulatory subunit type II) [30].

PGE_2_ has also been shown to inhibit AM FcR-mediated phagocytosis by activating the EP_2_ receptor, judged by the mimicked effect of the selective EP_2_ agonist butaprost [23] or a specific Epac-1 agonist (8-pCPT-2′-O-Me-cAMP) [32]. Moreover, PGE_2_ inhibits rat AM microbicidal activity and this effect was restored after treatment with indomethacin, EP_2_, and EP_4_ antagonists [31]. The role of EP_3_ receptor activation-driven AMs was also studied in the context of pulmonary infection. Although the G*α*_i_-coupled EP_3_ was thought to oppose the G*α*_s_-coupled EP_2_ and EP_4_ receptors, EP_3_^−/−^ mice were protected from bacterial induced death, which corroborates the increased ability of AMs to phagocytose and kills *Streptococcus pneumoniae *[33]. Through EP_2_, PGE_2_ was also involved in the mediation of the immunosuppressive response characterized by increased IL-10 synthesis and the impairment of neutrophil recruitment to the lungs during the ingestion of apoptotic cells (efferocytosis) by phagocytes [10]. As a suppressive mediator, PGE_2_ inhibits AA release and LTB_4_ synthesis in rat AMs by a mechanism independent of PLA_2_ [34].

Human and mouse lung DCs are localized in the airway epithelium, lung parenchyma, visceral pleura, and bronchoalveolar lavage fluid (BALF) [35]. DCs exposed to PGE_2_ exhibit a decreased capability to secrete proinflammatory cytokines [36]. They are in contact with many other cells in the lungs such as the airway epithelium, type II alveolar epithelial cells, AMs, pulmonary interstitial macrophages, (myo)fibroblasts, bronchus-associated lymphoid tissue (BALT) lymphocytes, nonadrenergic, noncholinergic (NANC) nerve endings, capillary endothelium, and mast cells. Although the particularly contribution of lung DC as producer of PGE_2_ is still unknown, there are several studies using bone-marrow-derived DCs (BM-DCs) showing that their immunomodulatory function is highly regulated by mediators including PGE_2_, potentially produced by neighboring cells in the lungs. BM-DCs exposed to PGE_2_ present decreased ability to secrete proinflammatory cytokines [36]. The importance of lung DC modulation by PGE_2_ is highlighted considering DC as the mediator cell of the adaptative immune response and the lungs as an important local tissue for airway microbial defenses [37].

 Lung PMNs are the primary cells recruited to the lungs during acute lung injury [38]. LPS is an important inducer of the inflammatory response by its activation of Toll-like receptor 4 (TLR4). After binding to TLR4, LPS triggers the synthesis of chemoattractants that induce PMN migration at sites of inflammation, such as the lung [39]. The overproduced PGE_2_ by lung PMNs from bone marrow transplantation mice is involved to the decreased ability of PMN to kill *Pseudomonas aeruginosa*, an effect restored by the PG inhibition with indomethacin [16]. However, evaluation of EP signaling in the PGE_2_-mediated impaired host defense by lung PMMs is much less appreciated.

Due to the low yield of murine alveolar macrophages, one plausible alternative to study PGE_2_ synthesis/actions is the use of alveolar macrophage cells lines. However, a very limited number of studies have been done to identify the profile of PGE_2_ synthesis and actions in this cell line. Here, we are summarizing some of the key findings regarding the expression of COX mRNA and protein in MH-S murine alveolar macrophages. MH-S is a murine alveolar macrophage cell line transformed by SV40 obtained from Balb/c mice and displays several properties of primary AM, such phagocytic capacity and expression of Mac-1 antigen, major histocompatibility complex class II, the CR3 receptor, and the Fc receptor Mbawuike and Herscowitz, 1989 to [40]. LPS-stimulated MH-S cell line promotes robust increment of COX-2 and large amounts of PGE_2_ (Joo et al., 2005 to [41]; Chen et al., 2007 to [42]). Luteolin, a flavonoid that exhibits anti-inflammatory properties, is shown to inhibit COX-2 gene expression and PGE_2_, IL-6, TNF-*α*, and iNOS production in LPS-activated MH-S cells by decreasing NF-*κ*B and AP-1 activation Chen et al., 2007 to [42]. In this context, LPS or overexpression of IKK*β* is reported to activate NF-*κ*B signaling and COX-2 expression, which was impaired after ectopic expression of hepatitis C virus in MH-S cells Joo et al., 2005 to [41]. However, so far there are no reports regarding EP receptors expression profile and the relative role of individual receptor in MH-S cells.

## 3. Spleen

Splenic macrophages, DCs, and lymphocytes contribute to PGE_2_ synthesis in the spleen [43]. In splenic tissues, mPGES-1 accounts for the majority of basal (COX1-dependent) PGE_2_ synthesis, and the *in vivo *mPGES-1 deletion abolished LPS-inducible PGE_2_ synthesis [44]. Normal splenic macrophages produce low levels of PGE_2_ when compared with bone-marrow-derived macrophages (BMDM; Table 1), AMs, and peritoneal macrophages [45]. However, high levels of this mediator are produced by splenic macrophages in chronic inflammatory conditions, such as mycobacterial infection [46]. It has been shown that the formation of PGE_2_-producing splenic macrophages is dependent on the radiosensitive bone marrow cells [47]; the precursors migrate from the bone marrow cells to the spleen to become mature cells [48]. Splenic DCs appear phenotypically immature and mature after microbial stimuli [37]. The phenotype seems to be determined by other suppressive mediators, including NO, TGF-*β*, 1*α*, 25 dihydroxyvitamin D3 (vitamin D) and PGE_2_ produced by antigen-presenting cells (APCs) such as macrophages and DCs [49]. To date, no reports have described EP expression in splenic DCs; most studies are focused on bone-marrow-derived DCs (BM-DCs) [50]. These cells express all four EP receptors [51] that can induce different effects, including DC generation, migration, and maturation [52].

PGE_2_-producing macrophages that are induced from mycobacterial stimuli interact closely with splenic lymphocytes to induce a shift from the Th1 to Th2 immune responses in a PGH_2_ synthase-dependent manner [53]. This shift is based on the suppressive effect of the synthesis of Th1 cytokines, such as IL-1, IL-12, and interferon (IFN)-*γ*, but it does not affect Th2 cytokines [54]. The downmodulation of TNF-*α* synthesis by PGE_2_ in *in vitro*-derived BM-DCs occurs through EP_2_- and EP_4_-induced signal transduction events [55]. It has also been shown that this signaling can upregulate IL-23 synthesis and downmodulate APC-produced IL-12 [56], which favors the expansion of IL-17-producing Th17 cells [57].

## 4. Bone

PGE_2_ produced in the bone is primarily derived from osteoblasts, cells responsible for bone formation [58]. As shown in Table 1, mouse BMDMs, osteoclast precursors, and mature osteoclasts differentially express EP receptors. BMDMs express the EP_1_, EP_2_, EP3*β*, and EP_4_ receptors, while mature osteoclasts only express the EP_1_ receptor [59]. It was demonstrated that PGE_2_ can stimulate cAMP levels in BMDMs but does not affect cAMP in mature osteoclasts; this result demonstrates that functional EP_2_ and EP_4_ receptors are inhibited in osteoclasts during its differentiation [59].

Osteoclasts are bone-resorbing multinucleated cells derived from the monocyte-macrophage lineage [60]. The differentiation and activation of osteoclasts are tightly regulated by osteoblasts through the release of receptor activator of NF-*κ*B ligand (RANKL) and macrophage colony-stimulating factor (M-CSF) [61], which are required for the differentiation of osteoclast progenitors into mature osteoclasts [62]. RANKL activation induces COX-2 expression in immature osteoclast by utilizing a Rac1-dependent NK-*κ*B activation pathway; that results in PGE_2_ synthesis and contributes to accelerated osteoclast differentiation [63].

In bone, PGE_2_ is known to be an important local factor in the regulation of bone formation [64] and resorption [65]. PGE_2_ acts in precursors and mature osteoclasts to regulate their function. PGE_2_ can directly inhibit the bone-resorbing activity of osteoclasts. This inhibitory effect was dependent on an increase of intracellular cAMP caused by activator of adenylate cyclase (forskolin) and mimicked by the EP_2_ and EP_4_ agonists (butaprost and AE-604). In calvaria culture from EP_4_ knockout mice, PGE_2_ presented an impaired role in promoting bone resorption, whereas EP_2_ agonist slightly restored bone resorption and EP_4_ agonist did not [66].

## 5. Central Nervous System (CNS)

Although the immunoprivileged status of the CNS is well known, similar to any other organ, it is connected and engaged with the immune system to maintain tissue homeostasis. An excessive inflammatory status can promote several types of brain damage, which include ischemia and neurodegenerative diseases, such as Alzheimer's disease and Parkinson's disease [67].

The CNS typically contains low prostanoid levels. Specifically, PGE_2_, PGD_2_, and PGF_2a_ are associated with inflammatory responses [68]. Oddly, the COX-1 and COX-2 enzymes are both constitutively expressed in the CNS (in neurons, astrocytes, microglia and endothelia) [69], and a putative COX-3 enzyme, which is a splice variant of COX-1 that is denoted as COX-1b, is described in rodent and human neural tissues [70–72]. The PGE_2_ levels in the CNS are enhanced during various neurological diseases, such as multiple sclerosis, Alzheimer's disease, and Parkinson's disease [68].

Importantly, the proinflammatory stimuli that lead to brain injury further enhance COX-2 expression and therefore enhance PGE_2_ synthesis. All three PGES isoforms are found in the CNS tissues, and the expression levels vary according to the cell type [73]. An elegant study demonstrated that brain PGE_2_ synthesis is orchestrated by COX-1/COX-2/membrane-associated cPGES (cPGES-m) and by nuclear/perinuclear COX-2/mPGES-1/cPGES [74].

Because few studies have described DCs and neutrophils in the CNS, we will focus primarily on the microglia functions. It is noteworthy that although there is a close relationship between the peripheral macrophages and microglia, all of the knowledge concerning the peripheral cells cannot simply be extended to microglia cells that are inserted in a unique environment.

Initially, astrocytes were reported to be the major source of prostanoids within the CNS [75], but later studies have demonstrated that microglial cells can release higher levels of PGE_2_, PGD_2_, and TXB_2_ than astrocytes [76]. Similar to peripheral macrophages, COX-2 is the main enzyme expressed by microglia after activation [77]. LPS induces high levels of PGE_2_ synthesis by upregulating COX-2 and mPGES-1 expression [76, 78]. Additionally, activation of microglia by TLR can be modulated by further PGE_2_ synthesis. Although factors such as TGF-*β* [79], TNF-*α* [80], norepinephrine [81], adenosine, and PGE_2_ [82], can act as COX-2 positive regulators, other factors, such as IFN-*γ* [83], IL-10 [79], NO [83], and lipocortin [84] are negative regulators of COX-2 expression and activation. Interestingly, PGE_2_ synthesis is rapidly augmented when microglia are treated with phosphatidylserine (PS) liposomes in a manner that is dependent on the COX-1/mPGES-2 axis [85].

From the moment that PGE_2_ is released, it acts in close proximity to its production site in an autocrine or paracrine manner. In general, PGE_2_ acts as a suppressive mediator of the microglia. In the CNS, PGE_2_ primarily causes enhanced levels of cAMP [80], which further suggests a role for EP_2_ and EP_4_ in the mediation of CNS inflammation. Supporting its suppressive functions, studies of TLR4-mediated microglial activation have shown that PGE_2_ can inhibit the production of TNF-*α* [86] and IL-12 [87], IL-18 [88], the expression of the B7-2 (CD86) co-stimulatory molecules [89], the enhancement of IL-10 and IL-6 production, and the expression of inducible nitric oxide synthase (iNOS). Additionally, a recent study has associated PGE_2_ with decreased microbicidal activity by microglial cells in meningitis [90].

In addition to its inflammatory roles, PGE_2_ is related to several central functions, such as fever (thermogenesis), the neuroendocrine axis, food intake, and behavior during sickness. Circulating IL-1*β* acts at the blood-brain barrier (BBB) to induce COX-2 expression and PGE_2_ synthesis, and PGE_2_ subsequently diffuses into the brain parenchyma to perform its actions [91]. Recent studies have revealed that central COX-2 inhibition did not abrogate fever induction or the increases in plasma corticosterones and anorexia, which suggests that other sources of PGE_2_, such as COX-2-dependent peripherally synthesized PGE_2_ or COX-1-dependent centrally produced PGE_2_ [92], are involved. Interestingly, PGE_2_ production in the spinal cord is elevated by peripheral inflammation through COX-2 and mPGES-1 induction, which is correlated with peripheral edema potentiation, enhanced neuron hyperexcitability, and hyperalgesia [93]. Moreover, COX-2-dependent PGE_2_ is an important signaling mediator for synaptic modification [94].

The role of PGE_2_ in the brain remains controversial, and its differential effects depend on its specific receptor [95]. Because the expression and timing of the EP receptors vary according to the cell type and neuronal stimuli, the specific role of each EP receptor depends on its specific context (for an extensive review, see [96]). The EP_3_ receptor is likely not associated with inflammatory roles, while the EP_2_ and EP_4_ receptors appear to have opposing activities [96]. Although the EP_2_ receptor is related to a proinflammatory neurotoxic effect in activated microglia [97], the EP_4_ receptor has an anti-inflammatory, neuroprotective role [98]. These contradictory effects reflect the differential expression and timing of the EP receptors.

Consistent with the myriad activities of PGE_2_ and the dependence on the expression of specific EP receptors in different cell types, studies that investigate the roles of PGE_2_ in the CNS should be addressed carefully. The inflammatory effects of PGE_2_ are related to its dual neuroprotective and neurotoxic roles, and unless the PGE_2_ paradoxical effects are finely tuned, neurodegenerative diseases could occur. A full understanding of the roles of PGE_2_ and the dynamics of EP receptors in the CNS requires the study of the restrained areas of the CNS and the endogenous PGE_2_ functions relative to the different cell types and receptors that are involved.

## 6. Reproductive Tract

Uterine macrophages are an important source of PGs for uterine activity [99]. They are known to be potent agonists that promote contractile activity in the uterus, and either PGs or its precursor treatments initiate preterm labor throughout gestation. Therefore, LPS-induced uterine activation may be due to increased levels of proinflammatory cytokine and PGE_2_. Furthermore, exogenously added PGE_2_ analogs can reduce the innate immune defenses within the reproductive tract. Slama et al. provided a good example of the role of PGE2 in inhibiting innate immune response. They injected a PGE_2_ analog into the maternal cervix of cows for 1 wk following calf delivery and observed an increased purulent uterine secretions, increased frequency and severity of bacterial contamination of the uterus, and reduced levels of antibodies in uterine secretions. Pharmacological PGE_2_ administration facilitated the establishment of chlamydial infections of the murine female reproductive tract [100]. We have shown that the intrauterine administration of misoprostol in rats infected with *Clostridium sordellii* further enhanced the bacterial numbers in the uterine tract and was followed by decreased animal survival. This effect was associated with the inhibition of TNF-*α* and defensin secretion by decidual macrophages and uterine epithelial cells [101]. Although little is known about the potential of misoprostol to suppress the reproductive tract's innate immunity, a study reported an increase in the rate of infections when misoprostol was administered orally, and the rate increased with intravaginal administration [102]. This may help to explain the connection between medical abortion and clostridial endometritis in contrast to infections that are caused by more commonly encountered pathogens.

## 7. Peritoneal Macrophages

Peritoneal macrophages are extensively used as a model to investigate macrophage function. This cell type is a standard model used to identify inflammatory responses, cellular metabolism, and apoptosis. Resident peritoneal macrophages exhibit low responsiveness to inflammatory stimuli relative to inflammatory peritoneal macrophages that are recruited by inflammatory stimuli, such as thioglycollate, peptone or glycogen. Resident peritoneal macrophages express mainly EP_4_ but not EP_2_ mRNA at basal levels. In the presence of LPS, the expression of EP_4_ mRNA is downregulated to levels that are lower than in nonstimulated macrophages, and the expression of EP_2_ mRNA is transiently increased after 3 h of stimulation [103].

Peritoneal macrophages have a greater capacity for PGE_2_ synthesis than macrophages from different organs, such as alveolar macrophages or spleen macrophages. These cells have higher levels of cytosolic and membrane COX-1 expression in activated cells, which are similar to the levels of COX-2 expression after LPS treatment [104].

The effect of PGE_2_ in the inhibition of inflammatory cytokines, such as TNF-*α*, IL-1*β*, and IL-6, was initially demonstrated in peritoneal macrophages upon TLR4 activation [105]. However, recent studies described that the effects of PGE_2_ are due to the production of IL-10 [106]. However, the suppressive effect of PGE_2_ on IL-6 production is controversial and seems to be dependent on the inflammatory stimulus used. In addition to the modulation of cytokines, exogenous PGE_2_ can also modulate the expression of the cell surface receptors of peritoneal macrophages. The addition of different concentrations of PGE_2_ induces an increase in CD14 on the surface of peritoneal macrophages through the activation of cAMP/PKA, which results in the activation of AP-1. The treatment of macrophages with a PKA inhibitor or with antisense c-fos and c-jun oligonucleotides in the presence of PGE_2_ prevented the increase of CD14 on the surface of these cells [107].

PGE_2_ modulates a broad range of cytokines in peritoneal macrophages involved in inflammatory processes. Endogenous PGE_2_ production in LPS-stimulated resident peritoneal macrophages acts as a brake for TNF-*α* and IL-12 synthesis [103]. The activation of peritoneal macrophages with other macrophage activators, such as IFN-*γ* and the fungal particle zymosan, induces the synthesis of cytokines, chemokines, lipid mediators, and reactive nitrogen and oxygen species that directly or indirectly modulate the synthesis of PGE_2_. Of the mediators that modulate PGE_2_ synthesis in these cells, NO seems to play a key role in inhibiting PGE_2_ biosynthesis by nitrosylating and preventing the activity of COX-2 and mPGES [108].

The capacity of PGE_2_ to modulate cytokine production clearly influences the inflammatory response during injury and infection. The susceptibility or resistance to infection in different mice strains could be associated, at least in part, with the ability to stimulate the production of eicosanoids from phagocytes. When they are stimulated with LPS, peritoneal macrophages isolated from Balb/c mice produce approximately 3-fold more PGE_2_ than the macrophages isolated from other mouse strains, such as C57BL. The higher levels of PGE_2_ in the peritoneal macrophages of Balb/c mice are associated with high expression levels of sPLA2 type V and mPGES mRNA relative to the levels in the macrophages of C57BL mice. The increased capacity to produce PGE_2_ by the macrophages isolated from Balb/c mice directly reflects the inhibition of cytokines, such as IL-12 and TNF-*α* [109].

The peritoneal site also represents a primary organ to generate macrophage cell lines, which are very often used to study macrophage behavior and functions. Below we will highlight some of the key human and murine cell lines used to study PGE2 production and actions.

## 8. RAW 264.7 Cells

RAW 264.7 cells are mouse macrophage-like cells established from the ascites of a tumor that was induced into a male Balb/c mouse by an intraperitoneal injection of Abselon leukemia virus (A-MuLV). These cells are extensively studied in models of inflammation, metabolism, and apoptosis, and they are used for *in vitro* drug screening. Currently, many reports have shown that EP_4_ is the most abundant EP receptor in RAW 264.7 cells, followed by EP_2_ and EP_3_ but not EP_1_ [110]. The expression of these receptors in RAW 246.7 cells can be modulated in a manner that is dependent on the inflammatory stimuli. TLR4 activation increases EP_2_ and inhibits EP_4_ receptor mRNA expression. In contrast, if these cells are stimulated only with IFN-*γ*, the expression of EP_2_ and EP_4_ decreases in a concentration-dependent manner [111].

Several inflammatory mediators, including TNF-*α*, IL-1 [112], and IFN-*γ* [113], can directly or indirectly increase the expression of COX-2 in RAW 246.7 cells. However, COX-2 expression and PGE_2_ synthesis in IFN-*γ*-treated RAW 264.7 cells is directly regulated by TNF-*α* [114]. In the presence of an inflammatory stimulus, PGE_2_ appears to have an autocrine effect in RAW 264.7 cells and can self-regulate the expression of COX-2. The pretreatment of cells with PGE_2_ or EP_2_/EP_4_ agonists followed by the stimulation with LPS induced an increase in COX-2 expression, and this expression was completely inhibited in the presence of an adenylyl cyclase inhibitor [115].

## 9. U937

U937 is a cell line isolated from the histiocytic lymphoma of a 37-year-old male and is used to study the differentiation of monocytes into mature macrophages in the presence of different stimuli, such as IFN-*γ*, phorbol 12-myristate 13-acetate (PMA), and vitamin D [116]. In PMA-differentiated cells, EP_4_ is the predominant receptor, while only low levels of EP_1_, EP_2_, and EP_3_ were detected [117]. Unstimulated U937s expressed high levels of EP2 on the surface; however, when these cells were incubated with different concentrations of PMA, the expression of EP_2_ and the cAMP levels that were induced by PGE_2_ decreased in a manner that was dependent on PKC [118].

Undifferentiated U937 cells produce low levels of PGE_2_; however, in the presence of 12-0-tetradecanoylphorbol13-acetate (TPA), these cells produce high levels of PGE_2_. U937 cells express high basal levels of PLA_2_, cPLA_2*α*_, and iPLA_2*β*_, and the presence of IFN-*γ* does not alter the expression of these proteins. The activation of these cells by the aggregation of Fc*γ*RI promotes the generation of PGE_2_, but only iPLA_2*β*_ appears to be involved in the release of AA and the generation of this prostanoid [119]. Untreated U937 cells or differentiated U937 cells in the presence of 1,25-dihydroxyvitamin D3 express only COX-1; however, when the differentiated cells are stimulated with serum-treated zymosan (STZ), they begin to express high levels of COX-2; in the presence of exogenous AA, they produce high levels of PGE_2_ [120]. U937 cells differentiated in the presence of PMA express COX-2 and high levels of PGE_2_, IL-1*β*, and TNF-*α* after 6 h of stimulation with LPS. However, unlike other cell types, the increased COX-2 levels in U937 cells are independent of the presence of IL-1*β* and TNF-*α* because the treatment of these cells with the respective neutralizing antibodies does not interfere with the expression of LPS-induced COX-2 [121].

## 10. Therapeutic Approaches

Because PGE_2_ is the major PG product of most organs and its synthesis is upregulated during inflammatory conditions, which include infections and pathophysiologic conditions, it is expected that PGE_2_ plays a nonredundant role in controlling the inflammatory response and modulating phagocyte function in diverse organs. Increased plasma PGE_2_ levels have been reported in murine models and in patients who have undergone bone marrow transplantation [16, 122], are infected with HIV [123], display protein-calorie malnutrition [124], are smokers, are aging [125], or have cancer [126] or cystic fibrosis [127]. In all circumstances, these conditions are associated with susceptibility to infection. More specifically, in a murine bone marrow transplantation model, high levels of PGE_2_ were observed in the lung and peritoneal lavage fluid, and the overproduction of PGE_2_ by multiple cell types, including AMs, PMNs, and alveolar epithelial cells, was observed [16]. Similarly, a bactericidal PMN defect in guinea pigs following thermal burn injury has been linked to increased intracellular cAMP levels and the overproduction of PGE_2_ [128]. In both a murine bone marrow transplant model and also a thermal burn injury, these defects were overcome by treatment with COX inhibitors. While COX inhibition is conventionally regarded to be an “anti-inflammatory” strategy, an alternative possibility is that COX inhibitors or other nonsteroidal anti-inflammatory drugs (NSAIDs) can prevent the overproduction of immunosuppressive PGE_2_, which may instead represent an “immunostimulatory” strategy. In contrast, in conditions in which PGE_2_ exerts proinflammatory activities, such as in arthritis, atherosclerosis, and fever, COX inhibition is also an attractive target due to its analgesic and antipyretic properties. These drugs also have the beneficial effects of pathogen clearance. This effect has been shown that the *in vivo* treatment with NSAIDs enhances microbial clearance in different models of infection [26]. Although it has not been explicitly tested, we speculate that PGE_2_ inhibition by NSAIDs should lead to reductions in intracellular cAMP levels, which may account for the immunostimulatory effects of NSAIDs in these models.

## 11. Conclusion

In summary, pharmacological inhibition or receptor genetic deletion in mice has unveiled the big diversity and distinct biological effects of PGE_2_. Depending on cell-specific signaling programs and the context of injury, EP receptors can mediate either bad or protective effects in processes that mediate various diseases. The development of highly selective pharmacological agents that targets individual EP receptors should be studied in clinical trials in different disease settings.

## Figures and Tables

**Table 1:** Prostaglandin E_2_ Synthesis and Receptor Expression in Leukocytes from different organs.

Type of compartment	Type of cells	Relative synthetic capacity	Receptor expression			
			EP_1_	EP_2_	EP_3_	EP_4_
	Neutrophils	−	+	+^&^	+	+^&^
Lung	Alveolar macrophages	+++	−	+++	+	++
	Dendritic cells	+*	+	++^&^	+	++^&^

	Neutrophils	−	ND	ND	ND	ND
Spleen	Macrophages	+*	ND	ND	ND	ND
	Dendritic cells	+	ND	ND	ND	ND

Bone	BMDM-derived	+ ++	+	+++	+	+++
	osteoclasts	+	+	++	+	++

Relative synthetic capacity is expressed by the number of plus (+) signs; a minus sign (−) characterizes no or a negligible synthetic capacity. Receptor expression is classified as positive (+), negative (−), minimal (±), or not determined (ND). *Synthesis of PGE_2_ is relatively low in unstimulated conditions but is upregulated upon stimulation. ^&^Receptor expression is upregulated during inflammatory stimulus.
